# Association of Diagonal Earlobe Crease with Risk of Atrial Fibrillation in Stable Patients with Coronary Artery Disease

**DOI:** 10.3390/jcm13185643

**Published:** 2024-09-23

**Authors:** Moo-Nyun Jin, Changho Song, Young Ju Kim

**Affiliations:** 1 Division of Cardiology, Ewha Womans University Medical Center, Ewha Womans University College of Medicine, Seoul 07985, Republic of Korea; 2 Division of Cardiology, BHS-Hanseo Hospital, Busan 48253, Republic of Korea; 3 Division of Cardiology, Shihwa Medical Center, Siheung 15034, Republic of Korea

**Keywords:** diagonal earlobe crease, atrial fibrillation, coronary artery disease

## Abstract

**Background**: Diagonal earlobe crease (DELC) is a proposed visible predictor of coronary artery disease (CAD). However, studies on the association between atrial fibrillation (AF) and DELC are lacking. This study evaluated the association between DELC and the incidence of AF in patients with CAD. **Methods**: A total of 669 participants aged <65 years (mean, 53.8 ± 7.5 years) diagnosed with CAD and without AF were evaluated for the presence of DELC. The study outcome was the incidence of AF based on the presence of DELC. The study period was planned for 60 months with a minimum follow-up period of 12 months. **Results**: Herein, the incidence of DELC was 10.8%. During the follow-up period (44.6 ± 14.9 months), the incidences of AF development were 16.4% and 8.4% in DELC and non-DELC groups, respectively. Kaplan–Meier analysis revealed that the occurrence of AF was significantly higher in the DELC group than in the non-DELC group (log-rank test, *p* = 0.02). Compared with patients without DELC, patients with DELC had a high risk of AF development (adjusted hazard ratio = 1.88, 95% confidence interval = 1.01–3.53). **Conclusions**: DELC is associated with an increased risk of AF in patients with CAD. These findings may aid in the detection of AF in patients with CAD.

## 1. Introduction

Diagonal earlobe crease (DELC) was first identified in 1973 by Sanders T. Frank and subsequently also termed “Frank’s sign” [1]. DELC is a diagonal fold or wrinkle that extends obliquely from the tragus towards the border of the earlobe [2]. DELC is considered to result from the loss of dermal and vascular elastic fibers [3]. Since its first description, many large prospective population-based studies have demonstrated that the presence of DELC is associated with an increased risk of ischemic heart disease and myocardial infarction, independent of age and other well-known cardiovascular risk factors [4]. DELC has been reported to be a predictor of major adverse cardiac events in patients with known coronary artery disease (CAD) [5,6]. Additionally, this visible sign has been associated with other cardiovascular risk factors. DELC is also associated with ischemic stroke [7]. The etiological relationship between DELC and atherosclerotic disease is not fully under-stood. DELC may occur because of microvascular disease-associated weakening of elastic fibers in the earlobes, reflecting a similar pathology in weakened coronary arteries [8,9].

DELC occurs more frequently with increasing age and is reported to be a simple visible dermatological marker for accelerated premature aging since it (1) is rare in children [10] (2) linked to telomere shortening [11], and (3) biomarkers of inflammation and oxidative stress are increased in patients with DELC [12].

Atrial fibrillation (AF) is the most common age-related heart rhythm disorder. AF and DELC are associated with common risk factors, such as hypertension, diabetes, obesity, hyperlipidemia, sleep apnea, metabolic syndrome, inflammation, oxidative stress, and aging [12,13,14]. DELC has been described as a useful dermatological marker for CAD. Both AF and CAD are common heart diseases and share common risk factors, with advanced age being associated with a higher risk of AF and CAD development [15]. Thus, we hypothesized that DELC may be a simple visible marker of both CAD and AF. However, studies on the association between DELC and AF are limited. The coexistence of AF and CAD worsens prognosis and makes management challenging for physicians [16]. Detection of AF in patients with CAD is clinically important. Therefore, this study aimed to assess whether the presence of DELC was associated with the incidence of AF. 

## 2. Materials and Methods

### 2.1. Study Population

The participants were consecutive patients aged <65 years who were diagnosed with CAD without AF and underwent coronary angiography between 2015 and 2020 in tertiary care hospitals in Seoul, Republic of Korea. CAD was angiographically defined as the presence of at least 50% luminal diameter stenosis in at least one major coronary artery [17]. In this study, CAD was confirmed by an interventional cardiologist using coronary angiography. The eligibility criteria were as follows: (1) patients aged <65 years who were diagnosed with CAD for the first time, and (2) patients who were judged to have stable angina on angiography, a stable clinical history, and managed with conservative medical therapy without revascularization. The exclusion criteria were as follows: (1) patients who had piercings or had suffered injuries or trauma to the ears, (2) patients with acute coronary syndrome, and (3) patients who had undergone open-heart valve surgery or coronary artery bypass grafting. Participants were prospectively recruited, and data were retrospectively analyzed. Written informed consent was obtained from all patients, and the study protocol was approved by our institutional review board.

### 2.2. Outcome and Follow-Up

The primary outcome was the detection of AF, which was defined as an episode of irregular heart rhythm without detectable P-waves lasting more than 30 s [18]. The detection was evaluated based on the standard 12-lead electrocardiogram (ECG) at 1, 3, 6, and 12 months and 24 h Holter monitoring at 6 and 12 months after enrollment. One year later, an ECG was performed every 6 months or whenever symptoms occurred. The planned study period was 60 months with a minimum follow-up period of 12 months.

### 2.3. Assessment of DELC

**Figure 1:**
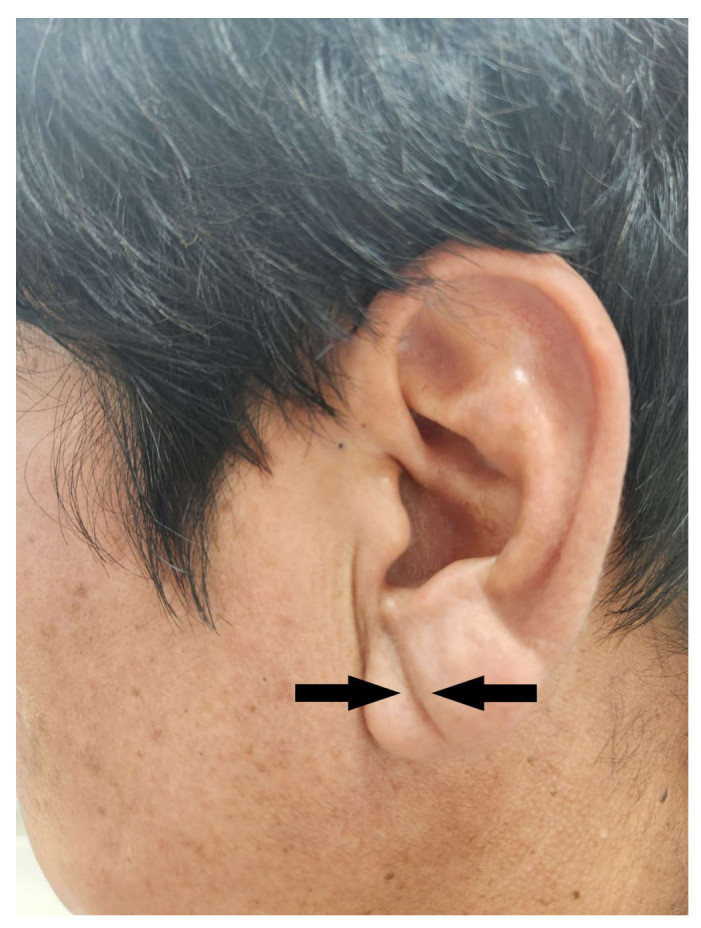
A typical example of a diagonal earlobe crease, indicated by arrows.

DELC was evaluated by taking photographs of both ears of the patients in the sitting position, which were then independently assessed for the presence of DELC by two trained doctors. DELC was defined in accordance with previous studies. Briefly, positive DELC was defined as a deep furrow or wrinkle extending diagonally from the tragus to-wards the outer border of the earlobe without a discontinuity. We considered both the unilateral and bilateral presence of such creases or wrinkles to be DELC-positive [19,20] (Figure 1). DELC was only assessed at baseline, and we did not investigate whether patients without DELC at baseline developed it during the follow-up period.

### 2.4. Statistical Analysis

Continuous variables are presented as means ± standard deviation or median as ap-propriate, while categorical variables are expressed as absolute numbers and percentages. To reduce any selection bias for treatment and other related potential confounding factors in this observational study, we performed a baseline characteristic adjustment for the participants using propensity scores. Propensity scores were estimated using a non-parsimonious multiple logistic regression model for the DELC and non-DELC groups. The following variables were considered based on their previously established roles as common risk factors in cardiovascular disease: age, sex, hypertension, diabetes, heart failure, prior stroke or transient ischemic attack, and dyslipidemia [21,22]. Pairs were matched in a 1:2 ratio using an optimal balance without replacement. A matching caliper of 0.2 standard deviations of the logic of the estimated propensity score was enforced to ensure that matches with a poor fit were excluded. Descriptive statistics were used to assess participants’ baseline characteristics and comorbidities. Continuous variables were compared using Student’s *t*-test or the Mann–Whitney U test, whereas categorical variables were compared using the chi-square test or Fisher’s exact test. Cox proportional hazards regression stratified by cohort was used to generate hazard ratios (HRs) and 95% confidence intervals (CIs) for the study outcomes. Models for clinical variable adjustments were used to assess associations. Multivariate models were adjusted for age, sex, heart failure, hypertension, diabetes, prior stroke or transient ischemic attack, chronic kidney disease, dyslipidemia, smoking, and alcohol intake, which were selected based on their previously established roles as predictive factors of AF [23]. Kaplan–Meier curves were generated using a log-rank test to show differences in primary outcomes according to the presence of DELC. Statistical significance was set at *p* < 0.05. Statistical analysis was performed using Statistical Package for the Social Sciences version 27 (IBM Corp., Armonk, NY, USA) and R version 3.6 (R Foundation for Statistical Computing, Vienna, Austria).

## 3. Results

### 3.1. Baseline Characteristics

A total of 669 CAD patients aged <65 years (mean age, 53.8 ± 7.5 years; 292 women) without a history of AF at baseline were included in the study. Of the 669 participants, 72 (10.8%) had earlobe creases. Baseline characteristics of the total and propensity score-matched populations are shown in Table 1. Patients in the DELC group were significantly older than those in the control group. Propensity score matching yielded a cohort that was well-balanced for all baseline covariates; there was no significant difference between the groups in terms of age, sex, comorbidities, and follow-up duration.

### 3.2. AF Development According to DELC

**Figure 2:**
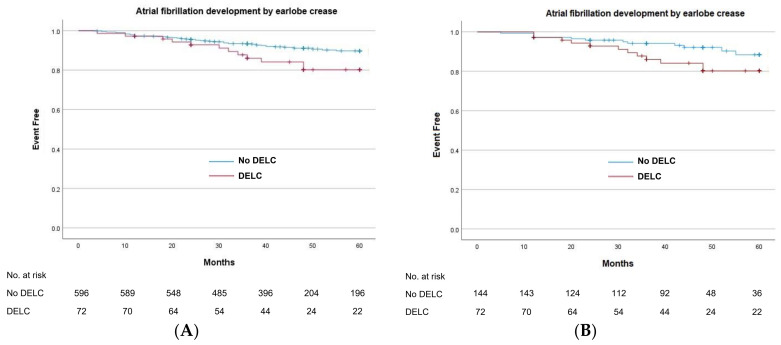
Kaplan–Meier curves for atrial fibrillation free survival rates in the total (**A**) and propensity score-matched (**B**) populations.

During the follow-up period (mean 44.6 ± 14.9 months), the development of AF was significantly higher in the DELC group (16.4%) than in the non-DELC group (8.4%) in the total population (*p* < 0.001). Similarly, in the propensity score-matched population, the DELC group showed a significantly higher incidence of AF than the non-DELC group (16.4% vs. 8.4%, *p* < 0.001). Kaplan–Meier analysis showed a significantly higher occurrence of AF in the DELC group than in the non-DELC group in the total population (Figure 2A, *p* = 0.02). This finding was confirmed in the propensity score-matched population (Figure 2B, *p* = 0.05).

In the multivariate Cox regression analysis, the presence of DELC was independently associated with a higher risk of AF occurrence (HR: 1.88 [95% CI, 1.01–3.53]; *p* = 0.04; Table 2). After performing propensity score matching, a trend in the association between the presence of DELC and AF development in the multivariate Cox model was observed (HR: 1.79 [95% CI, 0.91–3.49], *p* = 0.09, Table 2); however, it was not statistically significant. 

## 4. Discussion

The present study investigated the association between DELC and AF in patients with CAD. The main findings of this study were as follows: (1) patients with DELC and CAD had a significantly higher risk of AF than patients with CAD and without DELC. (2) The presence of DELC independently predicted the development of AF in patients with CAD. Our findings suggest that DELC might serve as a useful visible predictor of AF in patients with CAD. 

DELC, also known as “Frank’s sign”, is a wrinkle or fold line extending diagonally from the tragus towards the border of the earlobe. It was first associated with CAD by Sanders T. Frank in 1973 [1]. Most studies have confirmed the association between earlobe creases and the risk of atherosclerotic cardiovascular disease. DELC is a visible sign of coronary atherosclerosis [4]. DELC has also been associated with biomarkers of inflammation and oxidative stress [12]. Inflammation and oxidative stress are central to age-related cardiovascular disorders [11,24]; AF is the most common type of age-related arrhythmia. Both AF and DELC are associated with common age-related risk factors such as hypertension, diabetes, obesity, hyperlipidemia, and metabolic syndrome [23,25]. However, the usefulness of DELC as a risk marker for AF remains unclear. 

CAD is the most common type of cardiovascular disease, and AF is the most common type of arrhythmic disorder [26]. The incidences of both CAD and AF increase with advancing age, and concomitant CAD and AF are common in clinical practice. Previous studies have consistently demonstrated that patients with CAD who develop AF have an increased risk of all-cause mortality and cardiovascular events [27,28,29]. The management of concomitant CAD and AF requires close attention to avoid disease- and therapy-related complications. In particular, the strategies of antithrombotic agents and management goals may be concordant, and the attending physician must balance the risks of over- and under-treatment for each condition. Therefore, an important part of the management of patients with CAD is planning for AF detection during regular follow-ups. Although the chronic coronary syndrome guidelines recommend periodic 12-lead electrocardiography to assess heart rhythm and rate, there are no established guidelines for the identification of AF in patients with CAD [30]. Some studies reported that asymptomatic AF, which is a common phenomenon, was associated with a worse prognosis than symptomatic AF [31]; particularly, post-myocardial infarction asymptomatic new-onset AF was associated with poor long-term survival in the NOAFCAMI-SH registry [32]. Thus, earlier detection of AF might improve prognosis and facilitate the timely initiation of appropriate antithrombotic management. Identifying simple visible markers may improve the early detection of patients at risk of developing AF. DELC is a simple physical examination finding that may have prognostic value in patients with CAD [33]. The results of this study indicated that the presence of DELC in patients with CAD was associated with AF development. This may help raise suspicion and initiate screening for AF in patients with CAD. 

However, the mechanisms underlying the association between DELC and AF have not yet been investigated. Previous studies have shown that DELC is a marker for CAD, suggesting a common mechanism of microvascular changes related to subclinical atherosclerosis, as both the earlobe and myocardium are supplied by end arteries with few col-laterals, and these vessels easily become anoxic when obstructed [34,35]. There may also be a shared pathophysiology between DELC and AF, which is hypothesized to result from structural remodeling due to increased inflammatory and oxidative stress [12,36]. DELC has been associated with systemic inflammation and oxidative stress biomarkers, which are central to the pathogenesis of AF. The atrial remodeling process, involving fibrosis and electrical changes, is driven by these inflammatory processes, suggesting a possible mechanistic link between DELC and AF [14]. The presence of DELC may reflect a systemic state of heightened inflammation and oxidative stress, thereby identifying individuals at increased risk of AF. Both DELC and AF share common risk factors, including hypertension, diabetes, metabolic syndrome, sleep apnea, and aging [23,25]. Herein, DELC was significantly associated with the incidence of AF, even after adjusting for risk factors for CAD and AF. Further studies are warranted to determine the pathophysiological relationships and underlying mechanisms.

Limited data are available regarding the association between DELC and AF. To our knowledge, this is the first study to evaluate DELC as a predictor of AF, particularly in patients with CAD. However, this study has some limitations. This study was based on a single-center experience and was a retrospective, non-randomized controlled study, which could have introduced site-specific and selection biases. We reduced this bias using appropriate statistical methods. The medical therapy administered was determined by the attending physicians according to the characteristics of individual patients. However, the management was based on the same principles for individuals with similar risks. Additionally, DELC was evaluated only at baseline, and we did not examine whether patients who did not have DELC at baseline developed it during the follow-up period. This was because the primary focus of our research was on the association between the presence of DELC at baseline and the risk of AF. Future research involving long-term follow-up is needed to understand the potential changes in DELC and their relationship with AF development. A direct causal relationship between DELC and AF has not yet been clarified. Further studies are required to address these limitations.

## 5. Conclusions

DELC was associated with an increased risk of AF in patients with CAD. These findings may aid in the detection of AF in patients with CAD. Further studies are warranted to elucidate the pathophysiological relationship between DELC and AF and the suitability of DELC as a marker of AF. 

## Figures and Tables

**Table 1:** Baseline characteristics of the total and propensity score-matched populations.

Baseline Characteristics	Total Population			PS-Matched (1:2) Population		
	DELC Group (*n* = 72)	Non-DELC Group (*n* = 597)	*p*-Value	DELC Group (*n* = 72)	Non-DELC Group (*n* = 144)	*p*-Value
Age	55.6 ± 7.2	53.5 ± 7.5	0.03	55.6 ± 7.2	55.7 ± 7.2	0.91
Sex (Female)	26 (36%)	266 (45%)	0.17	26 (36%)	54 (37%)	0.84
BMI (kg/m^2^)	24.9 ± 3.1	24.8 ± 3.2	0.63	24.9 ± 3.1	24.7 ± 3.1	0.49
Smoker (former or current)	28 (39%)	191 (32%)	0.16	28 (39%)	50 (35%)	0.50
Alcohol (1 or more per week)	38 (53%)	280 (47%)	0.35	38 (53%)	76 (53%)	0.92
Follow-up period	43.3 ± 15.9	44.8 ± 14.9	0.44	43.1 ± 16.1	43.9 ± 15.9	0.72
Comorbidities						
Heart failure	8 (11%)	56 (9%)	0.64	8 (11%)	14 (10%)	0.88
Hypertension	41 (57%)	301 (51%)	0.30	41 (57%)	82 (57%)	0.92
Diabetes	13 (18%)	78 (13%)	0.24	13 (18%)	25 (17%)	0.96
Dyslipidemia	30 (42%)	239 (40%)	0.79	30 (42%)	60 (42%)	0.96
History of stroke/TIA	8 (11%)	58 (10%)	0.71	8 (11%)	14 (10%)	0.88
Chronic kidney disease	9 (12%)	65 (11%)	0.68	9 (12%)	17 (12%)	1.00
Vessels ≥ 50% stenosis by CAG			0.58			0.67
1	62 (86%)	528 (88%)		62 (86%)	125 (87%)	
2	8 (11%)	54 (9%)		8 (11%)	15 (10%)	
3	2 (3%)	15 (3%)		2 (3%)	4 (3%)	
Echocardiogram						
Left ventricular EF (%)	60.3 ± 10.1	61.4 ± 10.4	0.52	60.3 ± 10.1	60.7 ± 10.3	0.58
LAVI (mL/m^2^)	33.8 ± 11.4	32.1 ± 11.6	0.28	33.8 ± 11.4	33.2 ± 11.5	0.57
LA dimension (mm)	40.2 ± 6.9	38.7 ± 6.2	0.36	40.2 ± 6.9	39.9 ± 6.1	0.56

Values are expressed as n (%) or means ± standard deviations. BMI: body mass index, CAG: coronary angiogram, DELC: diagonal earlobe crease, EF: ejection fraction, LA: left atrium, PS: propensity score, TIA: transient ischemic attack, VI: volume index.

**Table 2:** Cox proportional hazards model for the prediction of AF development.

	Total Population				PS-Matched (1:2) Population			
	Univariate Analysis		Multivariate Analysis *		Univariate Analysis		Multivariate Analysis *	
	HR	*p*-Value	HR	*p*-Value	HR	*p*-Value	HR	*p*-Value
Age	1.07 (1.02–1.11)	0.002	1.05 (1.01–1.10)	0.026	1.07 (1.02–1.12)	0.009	1.06 (1.01–1.12)	0.033
Female	0.65 (0.38–1.10)	0.109			0.62 (0.33–1.17)	0.140		
BMI (kg/m^2^)	0.96 (0.89–1.04)	0.355			0.93 (0.84–1.03)	0.63		
Heart failure	1.72 (1.04–2.83)	0.078			1.43 (0.78–2.60)	0.241		
Hypertension	1.96 (0.79–4.89)	0.148			1.95 (0.27–14.28)	0.511		
Diabetes	1.12 (0.68–1.85)	0.653			1.11 (0.54–2.27)	0.784		
Dyslipidemia	1.25 (0.71–2.21)	0.443			1.25 (0.55–2.88)	0.596		
History of stroke/TIA	2.21 (1.18–4.16)	0.013	1.33 (0.64–2.79)	0.446	2.16 (0.96–4.85)	0.063	1.23 (0.49–3.11)	0.659
DELC	2.07 (1.10–3.88)	0.024	1.88 (1.01–3.53)	0.044	2.00 (1.02–3.88)	0.043	1.79 (0.91–3.49)	0.090

* Multivariate Cox analysis using statistically significant variables identified in univariate Cox analysis. BMI: body mass index, DELC: diagonal earlobe crease, HR: hazard ratio, PS: propensity score, TIA: transient ischemic attack.

## Data Availability

Data are available from the authors upon reasonable request with the permission of the Institutional Review Board of the Ewha Womans University Medical Center.
